# Type III Effector Activation via Nucleotide Binding, Phosphorylation, and Host Target Interaction

**DOI:** 10.1371/journal.ppat.0030048

**Published:** 2007-03-30

**Authors:** Darrell Desveaux, Alex U Singer, Ai-Jiuan Wu, Brian C McNulty, Laura Musselwhite, Zachary Nimchuk, John Sondek, Jeffery L Dangl

**Affiliations:** 1 Department of Biology, University of North Carolina at Chapel Hill, Chapel Hill, North Carolina, United States of America; 2 Department of Pharmacology, University of North Carolina at Chapel Hill, Chapel Hill, North Carolina, United States of America; The Rockefeller University, United States of America; 3 Department of Biochemistry and Biophysics, University of North Carolina at Chapel Hill, Chapel Hill, North Carolina, United States of America; 4 Lineberger Comprehensive Cancer Center, University of North Carolina at Chapel Hill, Chapel Hill, North Carolina, United States of America; 5 Department of Microbiology and Immunology, University of North Carolina at Chapel Hill, Chapel Hill, North Carolina, United States of America; 6 Curriculum in Genetics, University of North Carolina at Chapel Hill, Chapel Hill, North Carolina, United States of America; 7 Carolina Center for Genome Sciences, University of North Carolina at Chapel Hill, Chapel Hill, North Carolina, United States of America

## Abstract

The Pseudomonas syringae type III effector protein avirulence protein B (AvrB) is delivered into plant cells, where it targets the *Arabidopsis* RIN4 protein (resistance to Pseudomonas maculicula protein 1 [RPM1]–interacting protein). RIN4 is a regulator of basal host defense responses. Targeting of RIN4 by AvrB is recognized by the host RPM1 nucleotide-binding leucine-rich repeat disease resistance protein, leading to accelerated defense responses, cessation of pathogen growth, and hypersensitive host cell death at the infection site. We determined the structure of AvrB complexed with an AvrB-binding fragment of RIN4 at 2.3 Å resolution. We also determined the structure of AvrB in complex with adenosine diphosphate bound in a binding pocket adjacent to the RIN4 binding domain. AvrB residues important for RIN4 interaction are required for full RPM1 activation. AvrB residues that contact adenosine diphosphate are also required for initiation of RPM1 function. Nucleotide-binding residues of AvrB are also required for its phosphorylation by an unknown *Arabidopsis* protein(s). We conclude that AvrB is activated inside the host cell by nucleotide binding and subsequent phosphorylation and, independently, interacts with RIN4. Our data suggest that activated AvrB, bound to RIN4, is indirectly recognized by RPM1 to initiate plant immune system function.

## Author Summary

Many bacterial pathogens use a specialized protein “injection needle” called a type III secretion system to help colonize cells of higher organisms. The type III secretion needle attaches to a host cell and is the delivery conduit for a variety of bacterial proteins that act inside of the host cell. These proteins are called type III effectors. They manipulate host cell biology in order to help the bacterial pathogen colonize the host. We studied one type III effector from plant pathogenic bacteria called Pseudomonas syringae. This effector, termed avirulence protein B (AvrB), is targeted to the inner face of the plant cell plasma membrane, where it interacts with a membrane-bound host protein called RIN4 (resistance to Pseudomonas maculicula protein–interacting protein). RIN4 is phosphorylated in the presence of AvrB and an as-yet-unknown additional host factor. We provide a structural basis for the binding of AvrB to RIN4 and a possible mechanism of action for AvrB inside the host. AvrB activation and its ability to bind RIN4 have evolved to help the pathogen, yet in *Arabidopsis,* the AvrB-dependent phosphorylation of RIN4 is sensed by the plant immune system, leading to a rapid halt in pathogen growth.

## Introduction

Many Gram-negative bacterial pathogens of plants or animals employ type III secretion systems (TTSSs) to translocate type III effector proteins into host cells [1]. Type III effector proteins manipulate host cellular targets and signaling pathways to promote the infection process in genetically susceptible hosts [2,3]. In the plant immune system, specific nucleotide-binding leucine-rich repeat (NB-LRR) disease resistance proteins can monitor the homeostasis of type III effector targets [4–6]. In several cases, when a type III effector perturbs its target, the corresponding NB-LRR protein is activated. NB-LRR activation leads to a complex output including hypersensitive cell death (HR) and a suite of cellular responses that render the plant resistant to infection by pathogen strains expressing that type III effector.

The *Arabidopsis* NB-LRR protein RPM1 (resistance to Pseudomonas maculicula protein 1) recognizes the action of two distinct type III effector proteins, avirulence protein Rpm1 (AvrRpm1) and avirulence protein B (AvrB), which are found in various strains of the plant pathogen Pseudomonas syringae [7,8]. The RPM1-interacting protein (RIN4) is required for RPM1 function triggered by either AvrRpm1 or AvrB [9]. RIN4 physically associates in vivo with both AvrB and AvrRpm1, and with RPM1 [9]. The presence of either AvrRpm1 or AvrB in the plant cell leads to phosphorylation of RIN4, although neither of the type III effectors has sequence similarity to kinases [9]. RIN4 also interacts with, and is required for the function of, a second NB-LRR protein, RPS2 (resistance to P. syringae protein 2) [10,11]. The corresponding type III effector, avirulence protein Rpt 2 (AvrRpt2), is an autoprocessed cysteine protease that is activated by a host cyclophillin after delivery [12,13]. AvrRpt2 cleaves RIN4 at two sites [14,15], and the disappearance of RIN4 drives RPS2 activation [10,11].

One of the AvrRpt2 cleavage sites overlaps the AvrB binding site on RIN4 [15]. Thus, at least two independent type III effector proteins have evolved to target a small approximately 30–amino acid domain on RIN4 using at least two different biochemical mechanisms. It remains to be determined whether AvrRpm1 also targets this region of RIN4. This region is shared among several otherwise unrelated *Arabidopsis* proteins of unknown function and may represent a common motif targeted by plant pathogens [14,15]. RPM1, RPS2, the type III effectors, and RIN4 have been demonstrated, or are predicted to be, localized to the inside of the plant plasma membrane; AvrB, AvrRpm1, and RIN4 are acylated as a requirement for this localization [11,15–17].

Despite the wealth of genetic information for AvrB, its biochemical function remains elusive. A crystal structure of free AvrB revealed that it adopts a novel bilobal fold with no structural homologies to previously characterized proteins or functional domains [18]. A small upper lobe (amino acid residues 123 to 217) contains three alpha-helices (α5, α6, and α7) surrounding a five-stranded antiparallel beta-sheet (β1-β5-β4-β3-β2). A large lower lobe (residues 28 to 122 and 218 to 317) is composed strictly of alpha-helices [18]. The junction of the two lobes forms a large solvent-accessible cleft with a volume of over 900 Å^3^ that extends into the lower lobe to form a pocket rich in conserved residues. Chimeras of AvrB with the closely related paralog avirulence protein C (AvrC) (which does not activate RPM1) were used to demonstrate that the upper lobe of AvrB (residues 126 to 216) is required for RPM1 activation [18]. The lack of sequence conservation in the upper lobe among AvrB paralogs from various plant pathogens that do not trigger RPM1 mediated responses (see below) further supports this contention. In contrast, the lower lobe and the interlobal cleft are highly conserved in sequence among the AvrB family members. The conserved cleft was hypothesized to be an enzymatic active site that binds to substrate and/or cofactor [18].

In this study, we present the structure of AvrB bound to RIN4 and we identify interacting residues in the upper lobe of AvrB that are required for both RIN4 binding and activation of RPM1. We also identified a highly conserved nucleotide-binding pocket contained largely in the lower lobe of AvrB that is also required for activation of RPM1. In addition, phosphorylation of AvrB occurs in the presence of a host cofactor or kinase and may represent a third prerequisite for recognition by RPM1.

## Results

### Structure of AvrB Bound to RIN4

**Figure 1:**
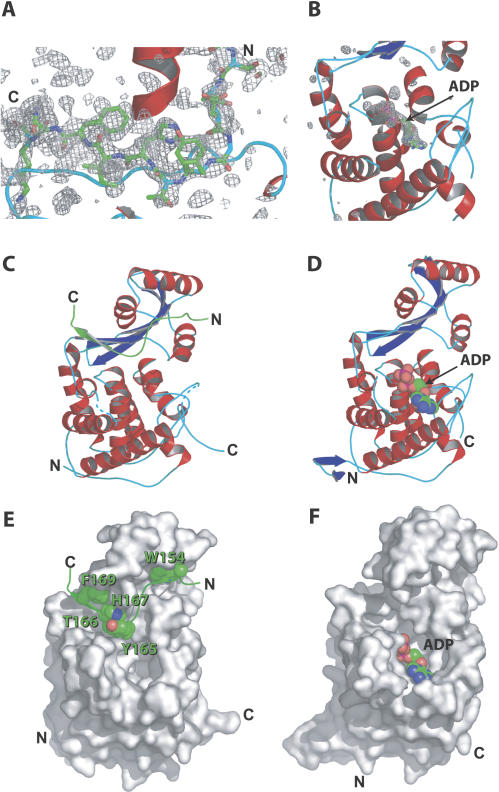
Structures of AvrB Complexed with RIN4_142–176_ and ADP (A) Difference electron density (*F*_o_ − *F*_c_) from AvrB/RIN4_142–176_ crystals centered on the C-terminal β-strand of RIN4_142–176_ (green sticks) following molecular replacement with two AvrB molecules (PDB 1NH1). (B) Difference electron density (*F*_o_ − *F*_c_) from AvrB crystals soaked with ADP following molecular replacement with 1 AvrB (PDB 1NH1). For (A) and (B), densities are contoured at 2 and 4 σ, respectively. (C and D) Ribbon diagrams of the AvrB/RIN4_142–176_ and AvrB/ADP complexes, respectively. AvrB helices, strands, and loops are red, blue, and cyan, respectively, and the RIN4_142–176_ ribbon is green. The overall structures are similar, and the different AvrB N and C termini between them likely reflects differences in their packing interfaces. (E and F) Surface representations of AvrB in the AvrB/RIN4_142–176_ and AvrB/ADP complexes, respectively. (E) RIN4_142–176_ is shown as a ribbon diagram with the side-chains of W154, Y165, T166, H167, and F169 represented as spheres to emphasize the surface grooves and concavities in AvrB that buries these residues. Note that the views represented in (C) and (E) and in (D) and (F) are identical.

**Figure 2:**
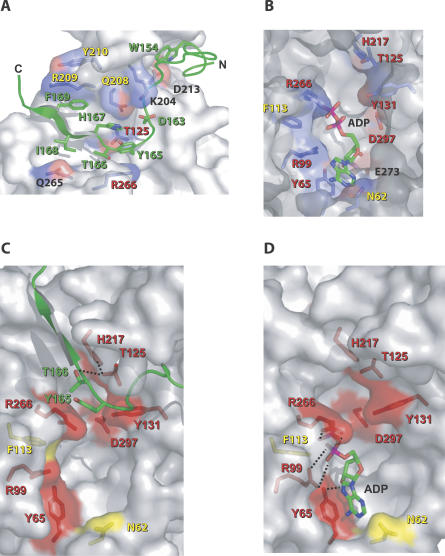
Close-Up Views of the AvrB/RIN4_142–176_ and AvrB/ADP Interfaces (A and B*)* A semitransparent representation of the AvrB surface showing selected AvrB side-chains that contact RIN4_142–176_ [green in (A)] and ADP in (B). Atoms of ADP and RIN4_142–176_ side-chains are colored by type: C (green), N (blue), O (red), and P (pink). AvrB side-chains are coded by atom type; C (light blue), N (blue), and O (red). Positive and negative surface charges of the indicated AvrB side-chains are color coded blue and red, respectively. Text for AvrB residues that were mutated and tested for their ability to trigger RPM1 function are color coded as follows: red, full loss of function; yellow, partial loss of function; black, no loss of function. (C and D) A close-in semitransparent representation of the AvrB surface highlighting the functional requirement (complete, red; partial, yellow) for AvrB amino acids in triggering RPM1 function. Relevant side-chains and surfaces of AvrB residues are displayed as stick representations with color-coded labels as in (A) and (B). Stick representations of residues from RIN4_142–176_ are labeled green in (C), and a stick representation of ADP is highlighted in (D).

Previous work using gel filtration and native gel electrophoresis demonstrated that AvrB bound a small RIN4 fragment consisting of amino acids 142 to 179 [15]. We cocrystallized AvrB with RIN4_142–176_ (Figure 1A, 1C, and 1E; Table 1). This fragment binds AvrB with affinity similar to that of the full-length protein (*k_d_* = 3 μM versus 4 μM as determined by isothermal titration calorimetry [ITC]; see below). Thus, essentially all of the binding energy for interaction with AvrB is contained in RIN4_142–176_. The structure of AvrB complexed to RIN4_142–176_ revealed that the peptide forms a “Z” shape with the N-terminal half contacting the upper lobe and the C-terminal half straddling the interface between the two lobes (Figure 1A, 1C, and 1E; Table 1). The interaction of AvrB with RIN4_142–176_ does not alter the overall structure of AvrB (unpublished data). Using this cocrystal structure, we identified residues of AvrB that interact with RIN4_142–176_ and could potentially be important for binding affinity (Figure 2A and 2C). The N-terminal half of RIN4_142–176_ interacts mainly through hydrophobic burial of RIN4 W154 plus a hydrogen bond between the indole nitrogen of RIN4 W154 and AvrB D213 (Figure 2A). RIN4 W154 is part of an AvrRpt2 cleavage site on RIN4 [14]. Additionally, AvrB T182 contacts RIN4 Y151, and AvrB V128 contacts RIN4 D155, S161, and G162.

**Table 1:**
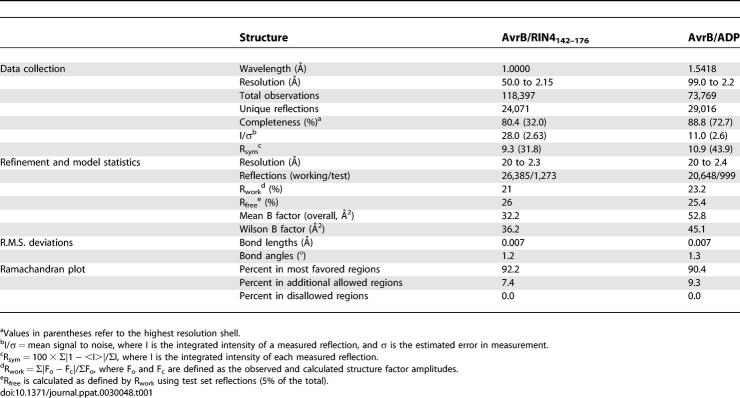
X-Ray Data Collection and Refinement Statistics

The most extensive interactions of RIN4_142–176_ with AvrB involve the C-terminal half of the RIN4 peptide. First, there is a set of antiparallel beta-strand hydrogen-bonding interactions between main-chain AvrB residues 120 to 124 (within strand β1) and RIN4 residues 167–171. Second, there is a hydrogen bond between the main-chain of RIN4 N170 and the AvrB R209 guanidinium group. These interactions allow this region of RIN4 to form an additional strand of the beta-sheet in the upper lobe of AvrB (Figure S1). Importantly, in forming this strand, the RIN4 T166 side-chain is directed toward the interior of AvrB such that the hydroxyl group hydrogen bonds with the hydroxyl group of AvrB T125, which in turn hydrogen bonds to the imidazole ring of AvrB H217 (Figure 2C).

Above this hydrogen-bonding arrangement, the aromatic rings of RIN4 Y165 and F169 as well as interleaving H167 are thrust against the surface formed from the last two turns of AvrB helix α7 (Figure 2A). This “ring-stack” of RIN4 spans from the AvrB upper lobe (R209) to the interlobe boundary (R266) (Figure 2A). These aromatic and indole side-chains stack in a nearly parallel arrangement with each other that is energetically very favorable. The guanidinium groups of AvrB R209 and AvrB R266 buttress the termini of the ring-stack, contacting RIN4 F169 and Y165, respectively, via π–π interactions such that the guanidium groups are also almost parallel to the three stacked aromatic rings of RIN4. In the middle of this ring stack, AvrB Q208 forms additional contacts with RIN4 H167 (Figure 2A). AvrB Y210 may play a weak role in RIN4_142–176_ binding since additional electron density from RIN4_142–176_ appears to be contacting this residue (Figure 1A). No additional electron density is observed for the last four residues of the RIN4 peptide (sequence REER).

### The C-Terminal Regions of RIN4_142–176_ Are Required for Binding to AvrB

**Figure 3:**
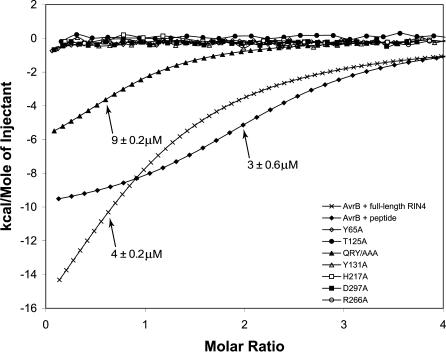
ITC Data for Binding of RIN4 to Wild-Type AvrB and Selected AvrB Mutants Fitted lines are shown for AvrB and mutants derivatives that did bind RIN4 (wild-type AvrB with full-length RIN4, wild-type AvrB with RIN4_142–176_ and AvrB^QRY/AAA^ to RIN4_142–176_). Raw data are displayed for AvrB mutants that did not bind RIN4_142–176_. Lack of binding was verified by using 5× to 10× concentrations of RIN4_142–176_ and AvrB variant relative to concentrations of wild-type components.

RIN4 and AvrB interact in yeast two-hybrid assays and can be coimmunoprecipitated from plant tissue [9]. This interaction is direct, because both purified proteins comigrate as an in vitro complex on native gel filtration columns [15]. To test whether the aforementioned AvrB residues are involved in RIN4 binding (listed in Table 2), we mutated the relevant amino acids individually to alanine. These mutants were assayed for their ability to bind full-length RIN4 by in vitro gel filtration and/or in vivo by yeast two-hybrid assay (Table 2). In addition, the binding affinity of selected AvrB mutants and RIN4_142–176_ was measured by ITC (Figure 3).

**Table 2:**
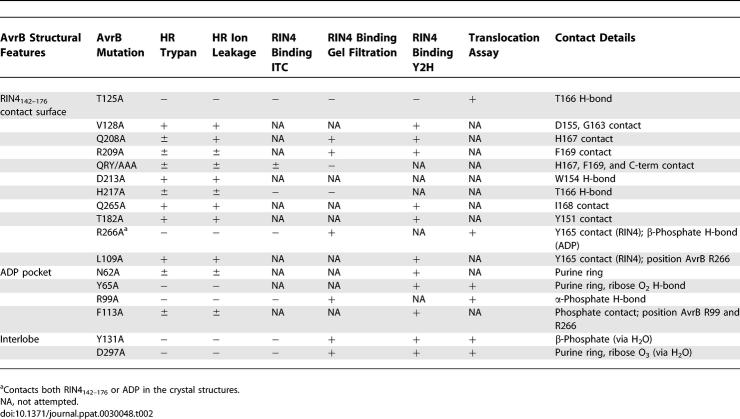
List of AvrB Mutations

The cocrystal structure revealed that the side-chains of several AvrB upper lobe residues make contacts with the N-terminal region of RIN4_142–176_. For example, D213 of AvrB interacts with W154 within the N-terminal portion of RIN4_142–176_. However, substitution of D213 to alanine did not disrupt either binding to RIN4 or the ability to trigger RPM1-mediated responses. The substitution of nearby residues of AvrB (i.e., V128A and T182A) also did not disrupt binding to RIN4 or activation of RPM1 (Table 2; unpublished data). These AvrB residues contact the N-terminal region of RIN4_142–176_ that contains an AvrRpt2 cleavage [14,15]. Hence, our data suggest AvrB and AvrRpt2 target two distinct amino acid sequences of this short, 35–amino acid residue region of RIN4.

In contrast, disruption of AvrB residues contacting the C terminus of RIN4_142–176_ strongly affected RIN4 binding. AvrB^T125A^ and AvrB^H217A^ were unable to bind RIN4_142–176_ under our ITC conditions (Figure 3). Loss of RIN4 binding activity was not due to significant structural disruptions, since AvrB^T125A^ or AvrB^H217A^ were properly folded, as measured by circular dichroism (unpublished data). Similar mutation of AvrB residues directly supporting the side-chains of the ring-stack also affected RIN4 binding. The single mutation AvrB^R209A^ had very little effect on RIN4 binding (Table 2) but a triple mutation of Q208, R209, and Y210 to alanine (hereafter AvrB^QRY/AAA^) significantly lowered the affinity for RIN4 (*k_d_* = 9 μM for AvrB^QRY/AAA^) and destabilized the AvrB/RIN4 complex (Figure 3 and Table 2). Overall, two regions of the upper lobe make distinct and functional contacts with the C-terminal region of RIN4_142–176_: (1) AvrB residues Q208, R209, and Y210, which directly support the side-chains of the ring-stack, and (2) AvrB residues T125 and H217, which interact with RIN4 T166, between Y165 and H167 of the ring-stack.

### RIN4-Interacting Residues of AvrB Are Required to Activate RPM1

**Figure 4:**
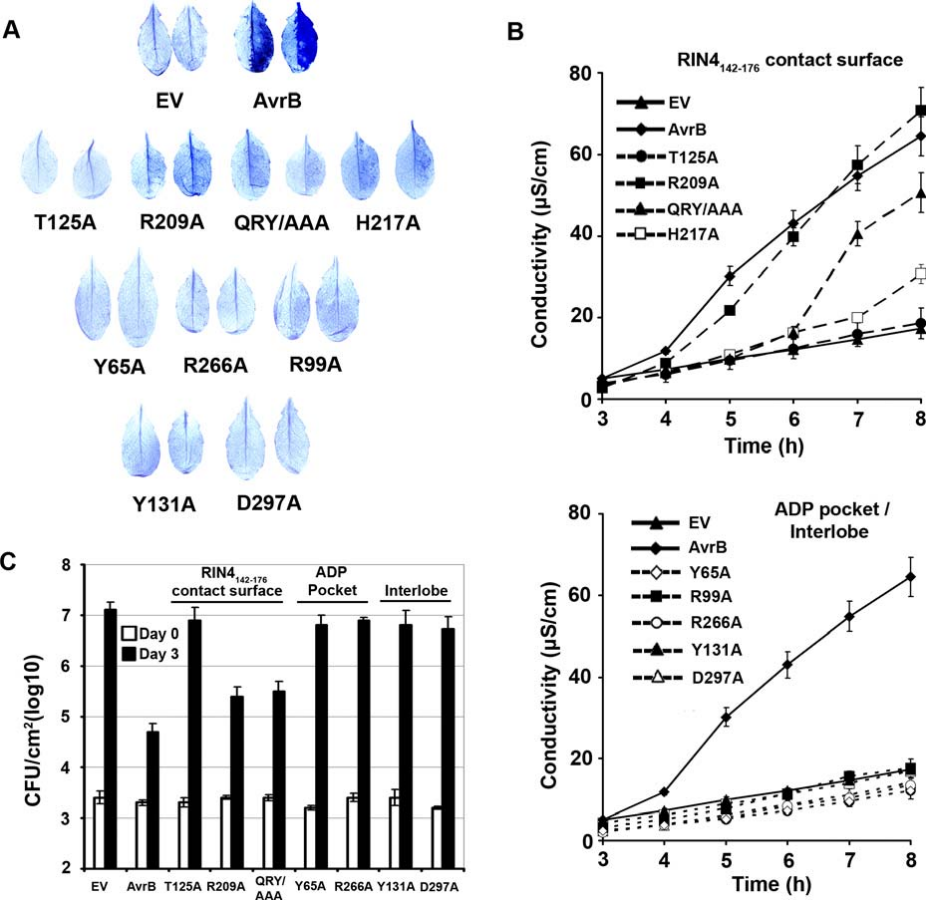
Interactions with RIN4 and ADP Are Required for AvrB Function in Activating RPM1 (A) Trypan blue staining of *Arabidopsis* Col-0 (*RPM1*) leaves 5 h after infection with *Pto* DC3000 expressing wild-type or mutant versions of HA-epitope tagged AvrB. EV is an empty vector (pBBR1 MCS-2) used as a negative control in *Pto* DC3000. QRY/AAA is a triple mutation of AvrB Q208, R209, and Y210 to alanine. Trypan blue staining of five infected leaves per construct was repeated twice in two independent experiments, and two representative leaves per AvrB mutant are presented. (B) Quantification of HR by electrolyte leakage (mean ± 2 SE) from leaves infected with *Pto* DC3000 expressing AvrB and mutant derivatives as labeled. The experiment was performed as in (A) and was repeated twice with similar results. Solid black lines represent the negative *Pto* DC3000(EV) (black triangles) and positive controls *Pto* DC3000(*avrB*-*HA*) (black diamonds). In the top graph, hatched lines represent mutations of AvrB residues making contacts with RIN4_142–176_ from the crystal structure presented in Figure 2. In the bottom graph, dotted lines represent mutations of AvrB residues lining the ADP binding pocket, from the crystal structure presented in Figure 2. (C) Growth of *Pto* DC3000 expressing the wild-type or selected alanine mutant versions AvrB-HA on *Arabidopsis* Col-0(*RPM1*). White and black bars represent the bacterial populations at the time of inoculation (day 0) and 3 d postinoculation (day 3), respectively. Bars indicate the mean of four samples ± 2 SE and are representative of two independent experiments.

AvrB induces phosphorylation of RIN4 [9], but it remained unclear whether AvrB must interact with RIN4 to trigger RPM1-mediated disease resistance and HR. We reasoned that if the RIN4–AvrB interaction is a prerequisite for RPM1-mediated HR or disease resistance, then AvrB mutants that do not bind to RIN4 should not trigger either phenotype. The preceding experiments identified AvrB mutants that varied in binding affinity to RIN4 (Figure 3 and Table 2). We expressed these *avrB* alleles in Pseudomonas syringae pv. *tomato (Pto)* DC3000. All of the AvrB alleles that exhibited altered function, as described below, were expressed at essentially normal levels in P. syringae (Figure S2D). *Arabidopsis* leaves were infected with *Pto* DC3000 strains expressing wild-type AvrB, each AvrB mutant, or an empty vector and assessed for RPM1-mediated HR using both trypan blue as a qualitative measure and the leakage of cellular ions into media and consequent changes in media conductivity over time as a quantitative measure (Figures 4A, 4B, S2A, and S2C). We also quantified the RPM1-mediated restriction of pathogen growth for selected AvrB mutants (Figure 4C). The results of these functional tests are summarized on the structures in Figure 2A and 2C.

V128, T182, and D213 are in a region of the AvrB upper lobe that is not required for interaction with RIN4 (Table 2). Unsurprisingly, these mutants have no effect on the ability of AvrB to activate RPM1 (Figure S2). The residues of AvrB that directly support the ring-stack of RIN4, especially R209 and Y210 (Figure 3 and Table 2), are more important for complex formation. Thus, while AvrB^R209A^ bound RIN4 (Table 2), and only marginally diminished RPM1-mediated HR in both assays (Figure 4A and 4B), disruption of several of the ring-stack interactions in AvrB^QRY/AAA^ drastically diminished RIN4 binding activity in vitro (Figure 3; Table 2) and resulted in significantly reduced RPM1-mediated HR (Figure 4A and 4B). These levels of altered HR correlated with slightly reduced RPM1-mediated restriction of bacterial growth. *Pto* DC3000*(avrB^R209A^)* and *Pto* DC3000*(avrB^QRY/AAA^)* grew in planta more than bacteria expressing wild-type *avrB* (Figure 4C).

Furthermore, mutations that disrupt the hydrogen bonding between AvrB T125 and H217 and RIN4 T166 (Figure 2A and 2C) eliminated or greatly reduced binding to RIN4 and the ability of these AvrB mutants to trigger RPM1 function (Table 2 and Figures 3 and 4). Translocation assays confirmed that AvrB^T125A^ was delivered into *Arabidopsis* cells (as are other loss-of-function AvrB alleles described here; Figure S3 and Table 2). Therefore, interactions that either directly or indirectly support the ring-stack of RIN4 are a major determinant for complex formation. Taken together, these results strongly suggest that binding of RIN4 by AvrB is a prerequisite for its ability to efficiently activate RPM1.

### AvrB Possesses a Nucleotide-Binding Domain Required for Function

The AvrB^R266A^ mutation was unable to trigger RPM1 function (Figure 4). The AvrB structure contains a cavity in the large lobe [18]. AvrB R266 lies at the interlobe boundary between the major RIN4-binding groove and this cavity (Figure 2). We hypothesized that this large cavity, rich in residues that are highly conserved across the AvrB protein family (see below), could serve as a binding site for a cofactor for AvrB activity. Because the presence of AvrB renders RIN4 marginally hyperphosphorylated [9], we wondered whether AvrB is an atypical kinase. We therefore examined binding to nucleotides, including adenosine triphosphate (ATP), adenosine diphosphate (ADP), guanosine diphosphate, and nonhydrolyzable ATP and guanosine diphosphate analogs, by soaking these into AvrB crystals. We found that crystallized AvrB could bind ADP within the lower lobe pocket of the large cavity (referred to as the ADP pocket; Figures 1B, 1D, 1F, 2B, and 2D; Table 1). We observed only weak density corresponding to other nucleotides in the lower lobe pocket (unpublished data). Significant interactions with ADP involve AvrB residues Y65, R99, and R266 (Figure 2B and 2D). Y65 stacks below the adenine ring and hydrogen bonds to the 2-OH group of the ribose, while R99 and R266 form salt bridges with the alpha- and beta-phosphates, respectively. N62 and F113 also contact the adenine base and phosphates, respectively (Figure 2B and 2D). F113 also appears to contribute to proper positioning of R99 and R266. The conserved AvrB residues Y131 and D297 located at the interlobe boundary are approximately 4 Å from ADP and appear to contact it only via bridging water molecules.

Mutation of ADP-binding residues in AvrB^Y65A^, AvrB^R99A^, and AvrB^R266A^ resulted in complete loss of AvrB-induced RPM1 function (Figure 4). Mutation of ADP-interacting residues AvrB^N62A^ and AvrB^F113A^ resulted in partial losses of RPM1-mediated HR (Figure S2B and S2C). Surprisingly, mutations that appear to contact ADP only via water bridges, AvrB^Y131A^ and AvrB^D297A^, also abrogated RPM1-mediated HR and bacterial growth restriction (Figure 4). Thus, all of the AvrB loss-of-function mutants in the ADP binding cavity eliminated the initiation of RPM1 function. We note, however, that they are each produced and properly folded (Figure S2D and circular dichroism, unpublished data) and translocated into host cells (Figure S3). Although AvrB^Y65A^ and AvrB^D297A^ failed to bind RIN4 in ITC (Figure 3), they, along with AvrB^R99A^, did bind RIN4 in yeast two-hybrid assay, gel filtration, or both (Table 2). We discuss these data further below.

### AvrB Is Phosphorylated in the Host Cell

**Figure 5:**
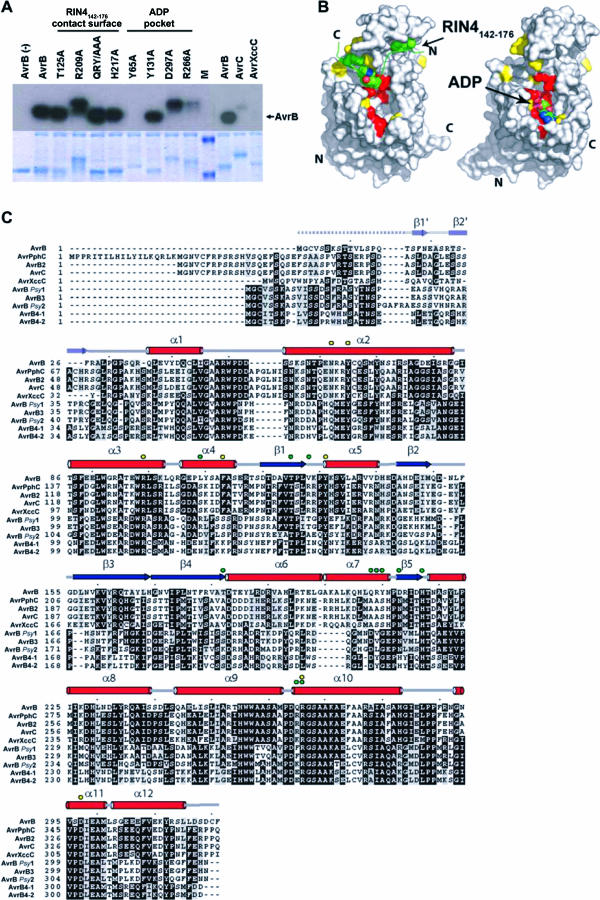
AvrB Is Specifically Phosphorylated in the Presence of *Arabidopsis* Extracts (A) Amino acids critical for AvrB function are required for its phosphorylation in the presence of *Arabidopsis* extract. Autoradiograph of reaction mixtures containing *Arabidopsis* (Col-0) extracts and [γ-^32^P]ATP with purified AvrB (36 kDa), indicated AvrB mutants, AvrB paralogs AvrC (39 kDa) and AvrXccC (36 kDa), or molecular weight markers (M). AvrB^R209A^, AvrB^D297A^, and AvrB^R266A^ include 14 additional amino acids from the recombination sequence of the Gateway cloning vectors at their C terminus (approximately 2 kDa). The lane labeled AvrB(−) does not contain plant extract. Coomassie brilliant blue staining of the radioactive gel, shown below, serves as a loading control. (B) Two surface representations of AvrB bound to RIN4_142–176_ (left) and ADP (right). Red and yellow patches represent full and partial loss of AvrB function with respect to triggering RPM1, respectively. RIN4_142–176_ is colored green, and amino acids making contacts with AvrB are highlighted (left). ADP is shown as a space-filled molecule (right). (C) AvrB residues contacting ADP are conserved among proteins of the AvrB protein family, but those contacting RIN4_142–176_ are not. Amino acid sequence alignment of ten AvrB paralogs. Helices, strands, loops, and unstructured regions are denoted by red tubes, blue arrows, gray lines, and dashed gray lines, respectively. Secondary structure elements from residues 16 to 27 are rendered transparent, as they have been observed in the AvrB/ADP, but not in AvrB/RIN4_142–176_ or free AvrB (PDB code 1NH1). Residues marked by green and yellow circles make contact with RIN4_142–176_ or line the ADP-binding cavity, respectively. The paralogs used in this alignment are from P. syringae pv. *glycinea* (AvrB, AvrC), P. syringae pv. *phaseolicola* (AvrPphC, AvrB2, AvrB4–1, AvrB4–2), Xanthomonas campestris pv. *campestris* (AvrXccC), P. syringae pv. *syringae* (AvrB *Psy*1, AvrB *Psy*2, AvrB3).

Since RIN4 phosphorylation is induced by AvrB, it was hypothesized that AvrB may possess kinase activity. However, we detected no AvrB-dependent phosphorylation of RIN4 or RIN4_142–176_ using in vitro radiolabeling experiments in the presence or absence of *Arabidopsis* extracts. We also found no evidence for in vitro autophosphorylation of AvrB (Figure 5A). We did, however, observe phosphorylation of AvrB in the presence of wild-type *Arabidopsis* extracts (Figure 5A). AvrB phosphorylation was sensitive to ethylenediaminetetraacetic acid (EDTA) and heat denaturation of the plant extract by boiling prior to assays, suggesting that AvrB phosphorylation requires cations and a heat-labile plant factor (unpublished data). Phosphorylation activity was specific for AvrB, the only member of this type III effector family demonstrated to trigger RPM1 function, since related paralogs (Figure 5C) did not or only weakly incorporated radiolabeled phosphate (Figure 5A). In this assay, AvrB phosphorylation appears to be RIN4 independent, as it occurs in extracts from *rin4 rpm1 rps2* mutant plants (unpublished data). Titrating increasing amounts of purified full-length RIN4 protein into extracts from *rin4 rpm1 rps2* did not alter the level of AvrB phosphorylation in vitro (unpublished data). Furthermore, phosphorylation of AvrB does not require RIN4 binding, since AvrB^QRY/AAA^_,_ AvrB^T125A^, and AvrB^H217A^ were readily phosphorylated (Figure 4A). These data show that RIN4 is not the plant cofactor that either directly or indirectly regulates AvrB phosphorylation in planta.

In contrast, AvrB residues that directly contact ADP are important for phosphorylation (Figure 5A). AvrB^R266A^ and AvrB^Y65A^ were partially and completely compromised for phosphorylation, respectively (Figure 5A). The requirement of ADP interacting residues for AvrB phosphorylation suggests that nucleotide binding by AvrB is critical for this event. On the other hand, AvrB^Y131A^ and AvrB^D297A^ were phosphorylated to the same levels as wild-type AvrB (Figure 5A). This correlates with the minor contribution of these residues to nucleotide binding observed from the crystal structure. Nevertheless, AvrB Y131 and D297 are required for the triggering of RPM1 (Figure 4), suggesting their involvement in another aspect of AvrB activation.

## Discussion

Our data significantly extend previous observations defining the key functional regions of the type III effector protein AvrB: its upper lobe and interlobal cleft mediate contact with the *Arabidopsis* target protein RIN4, and the lower lobe contains a pocket suitable for binding ADP or another similarly shaped molecule (Figure 5B). We defined three correlates for triggering AvrB-dependent, RPM1-mediated disease resistance function in *Arabidopsis*. These are AvrB's interaction with RIN4, its binding of nucleotide, or another small molecule of similar shape, and its likely phosphorylation in the presence of *Arabidopsis* extract.

Our structure-based functional analysis of the AvrB-RIN4**_142–176_** complex identified two main regions of interaction: AvrB T125 and H217 and AvrB Q208, R209, and Y210. These AvrB residues interact with bulky and aromatic RIN4 residues Y165, T166, H167, and F169 (previously termed the AvrB binding site [BBS] [15]) in a ring-stacking arrangement just C-terminal to the previously identified AvrRpt2 cleavage site (RCS2; [14,15]). Mutation of these AvrB residues interferes with the ability to trigger RPM1 function, demonstrating that physical interaction of AvrB with RIN4 is required for recognition by RPM1. AvrB residues Q208, R209, and Y210 are poorly conserved in other AvrB family members (Figure 5C), suggesting that it might be the specificity determinant for RIN4 binding.

The BBS and RCS are a functional, bipartite domain in RIN4 and approximately 11 additional proteins in *Arabidopsis* proteins (pfam05627). This RCS-BBS domain is widely distributed in multicellular plants evolutionarily distant from *Arabidopsis,* such as the moss Physcomitrella patens and the fern Cerapteris richardii (http://plantta.tigr.org) and thus may have conserved roles in the plant immune system. Within this domain, the RCS is highly conserved, while the BBS and the spacing between the RCS and the BBS is more variable. A number of other RCS-BBS proteins are cleaved by AvrRpt2 (which targets the RCS [14,15]), yet it remains to be seen whether the additional 11 BBS-containing proteins are also bound by AvrB. It is noteworthy that the N-terminal RCS-BBS domain of RIN4 does not bind AvrB [15], suggesting that the amino acid divergence between the N-terminal RCS-BBS and C-terminal RCS-BBS sequences of RIN4 is functionally relevant.

AvrB is also recognized by a second resistance gene, *Rpg1-b,* in soybean [19]. A random mutational analysis of AvrB identified nine individual amino acids required for induction of both RPM1-mediated HR on *Arabidopsis* and Rpg1-b function on soybean [20]. Among these, AvrB T125, Q164, and I215 were required for the HR phenotypes on both RPM1-expressing *Arabidopsis* and Rpg1-b–expressing soybean. All of these residues are in the AvrB upper lobe. The hydrophobic I215 lies underneath T125, and its mutation would likely disrupt the structural integrity of this region, and hence interaction with RIN4. Q164 is not solvent exposed and is also likely to disrupt AvrB structure when mutated. S268 is also in the lower lobe, and its substitution to isoleucine abrogated RIN4 binding in yeast two-hybrid assays [20]. The polar side-chain of S268 is partially buried and its mutation to a residue with a bulkier, nonpolar side-chain could have detrimental effects on the structure of AvrB, thus resulting in loss of RIN4 binding. A RIN4 ortholog is present in soybean and possesses an intact BBS domain. Whether this soybean RIN4 ortholog interacts with AvrB and is required for *Rpg1-b*–mediated resistance remain to be determined.

We also observed binding of ADP to the lower lobe pocket of AvrB (Figures 2 and 5B). Nucleotide contact residues (AvrB Y65, R99, and R266) are conserved between AvrB family members (Figure 5C) and are required for RPM1-mediated disease resistance responses. Thus, the ability of AvrB to trigger RPM1 function in *Arabidopsis* requires both interaction with RIN4 and an intact nucleotide binding pocket. Strikingly, Rpg-1b–mediated HR in soybean also required an intact AvrB nucleotide-binding pocket [20]. In these studies, AvrB^R266A^ did not elicit an HR in soybean. In addition, AvrB G46 and A269, which make contacts with ADP in our crystal structure, were also required for this activity in soybean [20].

**Figure 6:**
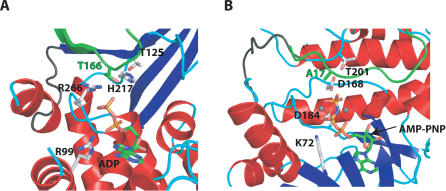
Functionally Important Residues of AvrB Correspond to Catalytic Residues in Ser/Thr Protein Kinases (A) A model of a possible ternary AvrB/RIN4_142–176_/ADP complex based on the crystal structures of AvrB/ADP and AvrB/RIN4_142–176_ (see Discussion). (B) The crystal structure of a ternary complex of cAMP-dependent kinase/inhibitor peptide/AMPPNP (PDB code 1CDK). The color scheme employed follows that of previous ribbon diagrams. RIN4_142–176_ and inhibitory peptide are shaded green in (A) and (B), respectively. Atoms of ADP and RIN4_142–176_ T166 in (A) and AMPPNP in (B) are colored by type: C (green), N (blue), O (red), and P (pink). Labeled residues are stick representations of those critical for AvrB/RIN4_142–176_ or AvrB/ADP interaction in (A), and those critical for S17 phosphorylation, inhibited by S17A, as shown in (B).

We hypothesize that AvrB binds to ADP, or another similarly shaped nucleotide, and interacts with RIN4 to induce RIN4 phosphorylation in *Arabidopsis*. If so, then AvrB might have a kinase or protokinase activity required to phosphorylate RIN4. Tertiary structure-matching programs such as DALI or MSD-Fold did not reveal significant structural similarity between AvrB and known kinases. However, the AvrB structure is similar to typical Ser/Thr protein kinases such as cAMP-dependent kinase, in that both contain a bilobal structure with a large lobe composed predominantly of alpha-helices and a small lobe with a mixed alpha-helix beta-sheet content [18]. We present a model of a ternary AvrB/RIN4/ADP structure created by superimposing the AvrB/RIN4 and AvrB/ADP coordinates and subsequent insertion of the ADP coordinates into the AvrB/RIN4 structure (Figure 6A). In this model, the distance between the oxygen atoms of the beta-phosphate of ADP and the T166 Oγ atom is quite short (4.2 Å), indicating that RIN4 T166 is a strong candidate for phosphorylation by AvrB.

We compared our modeled ternary complex to the structure of a ternary complex between cAMP-dependent kinase, AMPPNP, and an inhibitor peptide in which the acceptor serine 17 is replaced by an alanine [21] (Figure 6B). This comparison revealed that AvrB residues necessary to elicit an RPM1-dependent HR correspond in position to residues critical for the kinase activity of this cAMP-dependent kinase. Also, the positions of the phospho-acceptor in the inhibitor peptide and the putative acceptor T166 of RIN4 are similarly positioned. In cAMP-dependent kinase, the nucleotide binds in a cavity between the small lobe and large lobe, with the small lobe making up the majority of the interactions, whereas in AvrB the nucleotide binds in the major cavity of the large lobe. As a result, the orientation of the nucleotide is different between the two structures.

In cAMP-dependent kinase D168 deprotonates the acceptor serine/threonine residue and T201 positions D168 by hydrogen bonding. A similar role can be envisioned for AvrB H217 and T125, respectively, with regard to the putative acceptor T166 of RIN4. In addition, cAMP-dependent kinase K72 plays a role in phosphate binding and in stabilization of the transition state. It is analogous in position to R99 in AvrB. Although R266 in AvrB could also play this role, this residue is spatially most similar to D184 in cAMP-dependent kinase, which is involved in ligating a Mn^2+^ ion that chelates the terminal phosphate group. In the AvrB/ADP structure, we could not definitively observe a Mg^2+^ ion. The mechanism of metal binding in AvrB complexes remains to be elucidated.

Finally, protein kinases contain an activation loop joining the two lobes whose phosphorylation is necessary for protein kinase activity. In cAMP-dependent kinase, phosphorylation of T197 in the activation loop (residues 191 to 199) is necessary for kinase activity. In AvrB, residues 115 to 121 could form the activation loop, and three residues in this loop (T118, S119, and T121) could serve as phosphorylation sites. Additionally, this loop also borders the region (residues 120 to 125) that forms an antiparallel beta-sheet with residues 166 to 171 of RIN4. Hence, phosphorylation of this loop region may also affect RIN4 binding.

Despite this plausible similarity to kinases, pure AvrB neither autophosphorylates nor transphosphorylates pure RIN4 in vitro (unpublished data). However, we found that AvrB is phosphorylated in the presence of *Arabidopsis* extracts, suggesting that AvrB's possible kinase activity would require accessory plant factors. In vitro, this phosphorylation event is RIN4 and RPM1 independent. The P. syringae type III effector avirulence protein *Pseudomonas* tomato (AvrPto) is also phosphorylated in the presence of plant extracts independently of its corresponding plant disease resistance proteins, Pto and Prf [22]. Additionally, NopL and NopP are TTSS effector proteins from Rhizobium sp. NGR234 that are phosphorylated in the presence of protein extracts from L. japonicus [23,24]. Hence, there is a class of type III effector proteins that are phosphorylated once delivered to the host cell. We anticipate that this modification is linked to effector activation in all of these cases.

AvrB phosphorylation in the presence of *Arabidopsis* extract is dependent on the AvrB nucleotide-binding residues we defined, such as Y65 and R266. While nucleotide binding residues are required for AvrB to be phosphorylated, they are not sufficient, because AvrC and Xanthomonas campestris campestris avirulence protein (AvrXccC) encode the conserved nucleotide-binding region and are not phosphorylated in the presence of *Arabidopsis* extract. These data suggest that nucleotide bound to AvrB is required for the recruitment and/or function of a host kinase. Alternatively, AvrB could act as a “protokinase” that lacks intrinsic phosphor-transfer activity that can be enhanced by association with plant accessory protein(s). We speculate that each AvrB family member has evolved to usurp a plant species-specific protein that contributes to their activation by phosphorylation following delivery into host cells.

The ability of AvrB to trigger RPM1 function requires its nucleotide-binding pocket, which in turn is required for AvrB phosphorylation induced by an unknown *Arabidopsis* cofactor(s) or kinase, and interaction with RIN4. Neither AvrB^Y65A^, AvrB^Y131A^, nor AvrB^D297A^ nor bound RIN4 in ITC (Figure 3), although all three did in gel filtration and/or yeast two-hybrid assays (Table 2). The most parsimonious explanation for these binding data requires very slow association and dissociation rates of complex formation for AvrB and RIN4. For the wild-type proteins, these slow rates result in a modest equilibrium (*k*_d_ approximately 1 μM) measurable by ITC and sufficient for isolation of AvrB–RIN4 complexes when the two proteins are incubated together prior to gel filtration chromatography. Similarly, the two proteins would likely interact when coexpressed during yeast two-hybrid analysis. We propose, however, that AvrB^Y65A^, AvrB^Y131A^, and AvrB^D297A^ preferentially decrease the on-rate for AvrB–RIN4 complex formation. Hence, complexes could still form and be isolated by gel exclusion chromatography or inferred from yeast two-hybrid data due to the very slow off-rate. However, the resulting decrease in the overall equilibrium constant would prevent accurate affinity measurement by ITC. Implicit in this model is the likely requirement for a conformational change in AvrB that is slow and required prior to the binding of RIN4. Importantly, Y131 and D297 reside at the interlobal boundary of AvrB, and their substitution can easily be envisaged to shift the conformational equilibrium of AvrB and lock it in a state unfavorable for RIN4 association. This interpretation is consistent with the finding of Ong and Innes (2006) that AvrB^D297A^ enhanced binding of AvrB to RIN4 in their yeast two-hybrid system.

Our data are consistent with the activity of the AvrB protein family (Figure 5D). None of the AvrB homologs induce RPM1 function, although the Xanthomonas campestris protein AvrXccC interacts with RIN4 in yeast two-hybrid experiments and binds RIN4 weakly in vitro (unpublished data). Most of the AvrB-RIN4 contact residues are poorly conserved, particularly those in α6 and α7 helices, including AvrB Q208, R209, and Y210, which support the ring-stack of RIN4. For example, these AvrB residues correspond to AvrC residues A239, A240, and S241. AvrB T125, located on β1, is essential for RIN4 interaction and is conserved in all homologs of AvrB. Hence, the regions of AvrB that support the ring-stack of RIN4 appear to contribute to the functional specificity of AvrB for RIN4-dependent, RPM1-mediated HR. By contrast, the important ADP-contacting residues Y65, R99, and R266 in the lower lobe pocket are conserved in all AvrB homologs, suggesting that nucleotide binding is a core function for this entire type III effector family. However, an intact ADP binding cavity is not sufficient for phosphorylation of AvrB by *Arabidopsis* extracts, since the AvrC and AvrXccC possess them (including N62 and F113) but are not readily phosphorylated (Figure 5A). Furthermore, while AvrXccC interacts with RIN4, at least in yeast, and possesses the nucleotide-binding pocket, it does not trigger RPM1. This suggests that RIN4 interaction and nucleotide binding are not sufficient for the activation of RPM1 by AvrB family members.

AvrB Y131 and D297 are 4 Å away from the nucleotide binding site and are located in the solvent-exposed region of the interlobe cleft. Many of the solvent-exposed residues of the interlobe cleft are highly conserved within the AvrB family (Figure 5C). The electrostatic surface of the interlobe cleft and the sheer size of the cleft highly suggest that this might be an active site required for an as-yet-undefined activity of AvrB, or for the docking of an *Arabidopsis* protein that is required for AvrB phosphorylation. We speculate that the divergent sequences of the AvrB paralogs, centered on the interlobe cleft, are critical for recruitment of plant species-specific cofactors that enhance nucleotide turnover on each AvrB family member and/or align substrates of AvrB with potential catalytic residues in the cleft. We speculate that this set of atomic events would be required to trigger RPM1 (or other NB-LR) function in diverse plant species.

Besides acting as an avirulence factor to trigger resistance in *Arabidopsis* and soybean, AvrB can also serve as a virulence factor in susceptible hosts. In the absence of a functional *Rpg1-b* gene, AvrB enhances the growth of P. syringae on susceptible soybean cultivars [19] Similarly, AvrB also induces a cytotoxic yellowing response on *Arabidopsis* plants lacking *RPM1* that is attributable to either its function in virulence or a weak disease resistance response [17]. Mutation of AvrB T125, R266, or S268 to alanine abrogated the virulence phenotype in soybean and compromised the chlorosis phenotype in *Arabidopsis* [20]. Combined with our structural data, these results indicate that RIN4- and ADP-binding regions, as well as functions provided by the interlobe cleft, are required for the virulence activity of AvrB. Therefore, interaction with RIN4, nucleotide binding, and host phosphorylation are correlated with both the virulence activity of AvrB and for its recognition by independent plant NB-LRR proteins. This corroborates the “guard” model hypothesis where NB-LRR proteins monitor the activity of type III effectors for recognition rather than direct interaction [4,6].

Our three-dimensional crystal structures of AvrB in complex with its host target RIN4 or the ADP, and the combined functional studies detailed here and in Ong and Innes (2006), suggest a plausible series of events required for both AvrB virulence activity on susceptible hosts and for its ability to trigger disease resistance recognition in two plant species: AvrB is delivered by the TTSS of *Pto* DC3000. Inside the host cell, AvrB binds to a nucleotide, or another small molecule of similar shape. The AvrB–nucleotide complex recruits a plant cofactor that transforms AvrB into a kinase capable of autophosphorylation. Alternatively, the nucleotide-bound form of AvrB mimics a substrate for an unknown plant kinase and becomes phosphorylated itself. Because soluble AvrB can be labeled in an *Arabidopsis* extract, we infer that phosphorylation of AvrB is independent of, and hence might precede, myristoylation and plasma membrane localization [17]. Phosphorylated AvrB becomes myristoylated and directed to the plasma membrane. At the plasma membrane, AvrB interacts with RIN4, and its conformation is altered. This stable heterodimer guides RIN4 phosphorylation. The AvrB–RIN4 complex, and potentially the phosphorylated form of RIN4 itself, triggers RPM1-mediated activation of disease resistance. RIN4 functions as a negative regulator of basal defense [25]. Hence, in the absence of RPM1, AvrB is similarly activated and subsequently interacts with, and indirectly induces post-translational modifications of RIN4 and other BBS-containing proteins [15] in order to curb basal defense responses and contribute to disease.

## Materials and Methods

### Construction of clones and mutants.

For expression in *P. syringae,* AvrB-HA downstream of the AvrRpm1 promoter [17,26] was constructed by PCR amplification using primers that incorporated an XhoI site upstream of the AvrRpm1 promoter (XhoI-AvrRpm1p) and a BamHI site downstream of the HA epitope tag (BamHI-HA). The resulting PCR product was digested with the restriction enzymes XhoI and BamHI and cloned into the broad host range vector pBBR1 MCS-2 [27] digested with the same enzymes. Mutations of AvrB were generated by PCR using PFU turbo high-fidelity polymerase (Stratagene, http://www.stratagene.com). Overlapping primers incorporating the mutation of interest were synthesized, and PCR was conducted using the sense primer with BamHI-HA and the antisense primer with XhoI-AvrRpm1p. The resulting PCR products were gel purified, combined, and used as a template for a second PCR using the XhoI-AvrRpm1p and BamHI-HA primers. The resulting PCR product was cloned into the TOPO TA cloning vector (Invitrogen, http://www.invitrogen.com) and sequenced to ensure that no additional mutations had been introduced. The insert was then cleaved using the restriction enzymes XhoI and BamHI and cloned into pBBR1 MCS-2 digested with the same enzymes. pBBR1 MCS-2 containing the mutant AvrB-HA genes were then introduced into *Pto* DC3000 by triparental mating.

AvrB alleles from X. campestris pv. *campestris* strain 8004 (Xcc), P. syringae pv. *glycinea* race 0 (AvrC), and P. syringae pv. *syringae* strain B728A were PCR amplified from the corresponding bacterial strains and cloned into TOPO-TA (Invitrogen) cloning vectors (Z. Nimchuk and J. L. Dangl, unpublished data). To add an N-terminal glutathione-*S*-transferase (GST) tag, alleles were PCR amplified from TOPO-TA vectors using oligonucleotides that incorporated an N-terminal TEV cleavage site and subcloned into the pENTR D-TOPO vector (Invitrogen). The genes were then recombined into pDEST-15 vector using the LR Clonase enzyme mix according to manufacturer's instructions (Invitrogen). Similarly, mutated versions of AvrB were amplified from TOPO-TA and subcloned into pENTR D-TOPO followed by recombination into pDEST15 using LR recombination enzymes (Invitrogen).

### Protein expression and purification.

AvrB in pProEX-HTa was induced with 0.75 mM isopropyl-β-d-thiogalactopyranoside (IPTG) at 18 °C for 6 h in BL21 Rosetta cells (Stratagene). All protein purification steps were performed at 4 °C. Cell pellets were resuspended in buffer A (20 mM Tris [pH 8.0], 300 mM NaCl, and 10 mM imidazole) plus one “Complete EDTA-free” protease inhibitor tablet (Roche, http://www.roche.com), a few crystals of Lysozyme (Sigma, http://www.sigmaaldrich.com), and DNase (Sigma). After resuspension, cells were lysed using an Avestin Emulsiflex-C5 (Avestin, http://www.avestin.com) and centrifuged for 45 min at 15,000 rpm in an SS-34 rotor. The supernatant was loaded on to a 5 ml High Trap chelating column (GE Healthcare, http://www.gehealthcare.com) preloaded with nickel as described in the manufacturer's instructions. The column was then washed with 10 column volumes of buffer A, followed by 10 column volumes of buffer A augmented with 50 mM imidazole. Specific elution of AvrB was performed with 5 column volumes of buffer A containing 400 mM imidazole. Relevant fractions were pooled and dialyzed overnight at 4 °C in low-salt buffer containing 20 mM Tris (pH 8.0) and 100 mM NaCl in the presence of tobacco etch virus (TEV) protease to facilitate removal of N-terminal His tags. Removal of tags was verified by SDS-PAGE. The dialysate was loaded on an 8-ml Source Q (GE Healthcare) anion exchange column and eluted with a 0 to 400 mM NaCl gradient. If samples were not sufficiently pure at this point, relevant fractions were concentrated to approximately 10 ml and applied to a HighPrep 26/20 Sephacryl S200 (GE Healthcare) column equilibrated with 20 mM HEPES (pH 7.5), 150 mM NaCl, and 1 mM DTT. Purified protein was exchanged into 20 mM Tris (pH 8.0), 50 mM NaCl, and 3 mM DTT; concentrated to approximately 20 mg/ml and flash-frozen using liquid N_2_; and stored at −80 °C.

All AvrB mutants were cloned into the PD15 plasmid (GATEWAY; Invitrogen) and thus isolated as TEV-cleavable GST fusions. Induction was also performed with 0.75 mM ITPG at 18 °C for 6 h in BL21 Rosetta cells (Stratagene). Cell pellets were resuspended in buffer B (20 mM Tris [pH 7.5], 300 mM NaCl, and 1 mM DTT) and lysed as described for wild-type AvrB. Clarified lysates were loaded on a 5-ml High Trap glutathione column (GE Healthcare). The column was then washed with 10 column volumes buffer B, followed by specific elution by 3 to 5 column volumes of buffer B plus 10 mM glutathione. Eluted protein was digested overnight at 4 °C with TEV protease, and completely digested protein was diluted 5-fold, loaded on an 8-ml Source Q column, and eluted with a 0 to 400 mM NaCl gradient. Relevant fractions were then concentrated to less than 1 ml, flash-frozen in liquid N_2_ in approximately 250-μl aliquots, and stored at −80 °C.

RIN4 was cloned into pGEX-6P-1 as a GST-fusion cleavable with PreScission protease (GE Healthcare) and expressed in RIL codon-plus cells (Stratagene). Cells were grown to an OD of approximately 0.4 at 37 °C, and then the temperature was decreased to 25 °C for 45 min, and cells were induced for 3 h with 0.5 mM IPTG. Cell pellets were resuspended in buffer C (20 mM sodium phosphate [pH 6.5], 2 mM DTT, 1 mM EDTA) plus one “Complete EDTA-Free” protease inhibitor tablet (Roche), a few crystals of Lysozyme (Sigma), and DNase (Sigma). Cells were lysed as described for AvrB. Clarified lysates were then loaded on a hand-poured 20-ml Fast Flow S (GE Healthcare) column, washed with low-salt buffer, and then eluted with a 20 column volume gradient of buffer C plus 0 to 500 mM NaCl. The resulting broad peak was concentrated to 50 ml, 50 units/ml PreScission protease was added, and the mixture was dialysed overnight into buffer D (20 mM HEPES [pH 7.5], 50 mM NaCl, 2 mM DTT). Completely digested protein, as verified by SDS-PAGE, was then loaded on a 8-ml Source S column (GE Healthcare) and eluted with a 0 to 400 mM NaCl gradient. Relevant fractions were then pooled and again dialyzed overnight in buffer C. The dialysate was then run on a 8-ml Source Q column (GE Healthcare) and eluted with a 0 to 400 mM NaCl gradient. The purity of the samples was verified by SDS-PAGE, concentrated to approximately 2.5 mg/ml, flash-frozen in liquid N_2_ in approximately 250-μl aliquots, and stored at −80 °C.

### Gel filtration.

Gel filtration experiments of the mutant AvrB proteins were performed by loading approximately 0.3 to 0.5 mg of protein either alone or with roughly equimolar RIN4 in a volume of 1 ml onto a hand-poured calibrated 16/70 Superdex S-75 column. Flow rate was 0.9 ml/min, and 3-ml fractions were collected from 30 to 80 ml over a 150-ml run.

### Circular dichroism.

Circular dichroism experiments were run on a Pistar-180 Circular Dichroism/Fluorescence spectrophotometer (Applied Photophysics, http://www.photophysics.com). Samples at approximately 0.1 mg/ml were exchanged into a buffer containing 20 mM potassium phosphate (pH 7), and placed in a 0.1-cm cuvette, and scans were taken from 185 to 260 nm with 0.2-nm increments and 30,000 repetitions per increment.

### Isothermal titration calorimetry.

ITC experiments were performed on a VP-ITC microcalorimeter (MicroCal, http://www.microcalinc.com). To verify binding, we used concentrations of wild-type and variant AvrB ranging between 5 and 25 μM AvrB. RIN4_142–176_ peptide with concentrations ranging from 50 to 450 μM was titrated in 6-μl injections with stirring at 255 rpm. Experiments involving wild-type AvrB and full-length RIN4 used 6 μM and 120 μM, respectively. Once binding was confirmed (for wild-type and QRY/AAA), experiments were repeated in triplicate. Nonbinding was confirmed by increasing the concentrations of both the proteins to as much as 25 μM and 450 μM, respectively. Nonbinding variants were confirmed by repeating the experiment twice. Thermodynamic parameters were fit to the data using Origin v 7.0383 software (OriginLab, http://www.originlab.com).

### Crystallization.

Conditions for crystallization of AvrB and AvrB/RIN4_142–176_ were similar to those reported for AvrB previously [18]. Free AvrB was crystallized by vapor diffusion at 4 °C of a 1:1 mix of protein (8 to 10 mg/ml) with well solution (100 mM glycine [pH 9.0], 20% to 30% polyethylene glycol 550 monomethyl ether [PEG 550 MME]). AvrB/RIN4_142–176_ was also crystallized by vapor diffusion at 18 °C of an approximately 1:2 to 3 ratio of protein to peptide, mixed with an equal volume of well solution (100 mM Tris [pH 7.5], 20% to 30% PEG 550 MME). Initial crystals were of poor quality and were heavily twinned. This crystalline mass was resuspended in 50 μl of well solution, broken up and serially diluted 10-, 100-, and 1,000-fold, and used for microseeding. In general, seeds yielded suitable quality crystals within 2 d. AvrB/RIN4_142–176_ crystals belonged to space group P2(1) with cell dimensions a = 45.9 Å, b = 58.2 Å, c = 119.8 Å, and β = 89.9°, which corresponds to a very different packing than the P6(5) crystals found for crystals of free AvrB [18]. Soaks of nucleotide were performed by exchanging drop and reservoir solutions with 20 μl of 27% PEG 500 MME and 100 mM Tris 7.5 (with and without 5 mM MgCl_2_), followed by a final exchange in the drop of this solution plus 5 mM nucleotide. Nucleotides were soaked for approximately 1 d. Following soaks, crystals were found to pack in the P6(5) space group, with cell dimensions a = b = 122.7 Å, c = 64.1 Å, which is within 2.5 Å of the cell dimensions reported for free AvrB [18]. All crystallization solutions described in this section are inherently cryoprotective, and no further cryoprotection was found to be necessary.

### Diffraction and structure determination.

AvrB/RIN4_142–176_ diffraction data were collected on a Rigaku RU-H3R (http://www.rigaku.com) rotating anode generator equipped with Osmic confocal “blue” optics, and diffraction intensities were recorded on an R-Axis IV++ image plate system. For the ADP-soaked crystals, diffraction data were collected at the SER-CAT beamline (ID-22; Advanced Photon Source, http://www.aps.anl.gov). All structures were solved by molecular replacement using AMoRe [28] using the previously solved free AvrB structure (PDB code 1NH1) [18]. Upon molecular replacement followed by rigid-body refinement, simulated annealing, and individual B factor refinement using CNS [29], difference electron density could be found for both the peptide and nucleotide (see Figure 1A and D). Peptide and nucleotide were then modeled into the resulting difference density using the program O [30]. Definitions of ADP torsions in O, as well as topology and parameter files for all nucleotides, Tris, and trifluoroacetic acid, were taken from the Hic-Up server (http://xray.bmc.uu.se/hicup). This was followed by iterative cycles of simulated annealing, B factor refinement, and water picking to reach the results shown in Table S2. In addition, restrained 2-fold noncrystallographic symmetry was used during refinement of the AvrB/RIN4_142–176_ structure. From two AvrB/RIN4_142–176_ complexes in the asymmetric unit, 5,389 atoms are modeled, including residues 26 to 53 and 56 to 321 of AvrB and residues 150 to 172 of RIN4, in both complexes. In addition, 325 water molecules, four Tris molecules, and two trifluoroacetic acid molecules were included in the model. For the AvrB/ADP structures, there are 1,503 atoms, including residues 16 to 319 of AvrB, 1 nucleotide, one Tris molecule, and 82 water molecules.

### Western blot analyses.

For Western blot analyses, 1.5-ml overnight cultures grown in KB with the appropriate antibiotics were pelleted, washed with *hrp* gene-inducing minimal media [26], and resuspended to an OD_600_ of 0.1 in minimal media. Then, 2.5 ml of the 0.1 OD_600_ culture was induced overnight and spun down the next day. Pellets were resuspended in 250 μl of 20 mM Tris/HCl (pH 8.0) and sonicated twice for 10 s with a 1-min interval between. The sonicated culture was spun down at 4 °C for 20 min at 20,000*g*. Then, 200 μl of the supernatant was removed carefully so as not to disturb the pelleted and centrifuged again at 4 °C for 20 min. Next, 150 μl of the supernatant was carefully removed and soluble protein quantified. And 20 μg of protein of soluble protein from the wild-type and mutant AvrB-HA expressing *Pto* DC3000 strains was loaded onto SDS-PAGE gels after equalizing volumes with 20 mM Tris/HCl (pH 8.0), 6× Laemmli buffer was added, and it was boiled for 5 min. Immunodetection was performed by standard methods using anti-HA antibodies (Roche) at a dilution of 1:1,000.

### Stains and cell death quantification.

Lactophenol–trypan blue was used to visualize dead cells 5 h postinoculation with *Pto* DC3000 expressing wild-type and mutant AvrB-HA constructs as previously described [31]. Electrolyte leakage assays were carried out as previously described [31]. Briefly, fully expanded leaves from 3-wk-old plants were hand-inoculated with 0.1 OD_600_ (approximately 5 × 10^7^ cfu/ml) *Pto* DC3000 DC3000 expressing wild-type or mutant AvrB-HA constructs. At 2 h after infection, 7.5-mm leaf discs were collected and washed extensively with distilled water for 1 h. Four leaf discs were placed in a tube with 6 ml of distilled water (four replicates per treatment), and conductivity was measured over time with a conductivity meter (model 130; Orion Research, http://www.thermo.com).

### Kinase assays.

Plant extracts were prepared by grinding two or three 2-cm^2^ leaves to a fine powder in liquid nitrogen using a mortar and pestle. Then, 1 ml of grinding buffer (20 mM Tris/HCl [pH 8.0], 50 mM NaCl, 0.01% Triton X-100, 5 mM DTT) was added to the powder in a 1.5-ml microcentrifuge tube, and the mixture was vortexed for 30 s, followed by centrifugation for 10 min at 2,000*g* to remove large cell debris. The supernatant was collected and used as total plant extract. The 20-μl reactions contained 100 ng of purified AvrB allele or AvrB mutant, 1 μg of plant extract, 10 μM [γ^32^P]ATP (1.2 μCi; Amersham Biosciences, http://www.amershambiosciences.com), 100 μM ATP, and 10 mM MgCl_2_. Reactions were allowed to proceed for 10 min and terminated by adding 5 μl of 5× Laemmli buffer and boiling for 5 min. Reactions were loaded onto 12% SDS-PAGE gels, and incorporated radiolabel was visualized by autoradiography. As an equal loading control for proteins used in the kinase reactions, the SDS-PAGE gels were stained by Coomassie blue after detection.

### In planta growth assays.

*Pto* DC3000 strains containing the wild-type or mutant AvrB-HA constructs were streaked out onto King's medium B (KB) plates containing the appropriate antibiotics and incubated at 28 °C overnight. Bacteria were then scraped off the plate and resuspended to an OD_600_ of 0.0002 (approximately 1 × 10^5^ cfu/ml) in 10 mM MgCl_2_. Three-week-old plants were hand-inoculated with the diluted bacterial solution. Each sample was collected in quadruplicate using four leaves for each time point (16 discs per time point). Leaf discs were bored from the infiltrated area, ground in 10 mM MgCl_2_, and serially diluted to quantify bacterial numbers.

### LexA yeast two-hybrid.

For yeast two-hybrid analysis, *avrB* and its mutant derivatives were cloned into the Gateway-compatible LexA binding domain (BD) fusion vector pEG202 using LR Clonase II enzyme mix (Invitrogen). The LR reaction was left to proceed overnight at 16 °C. Yeast two-hybrid analysis was performed using the MATCHMAKER LexA system (Clontech, http://www.clontech.com) following the manufacturer's protocols. The yeast strains used in this study are EGY48 (Clontech) and RFY206. RIN4 was expressed from the plasmid pJG4–5 as B42 activation domain (AD) fusions and transformed into yeast strain EGY48 (MATα). *avrB* and its mutant derivatives were expressed from plasmid pEG202 and transformed into the yeast strain RFY206 (MAT a) carrying the lacZ reporter plasmid pSH18–34 (+pSH18–34). Preparation of highly competent yeast cells and small-scale lithium acetate transformations were performed using the Frozen-EZ Yeast Transformation II Kit (Zymo Research, http://www.zymoresearch.com). The RFY206 (+pSH18–34) transformants carrying pEG202:*AvrB* and its mutants were selected on minimal SD glucose agar base [0.7% yeast nitrogen base without amino acids and 2% bacto-agar supplemented with –Ura–His Dropout (DO)] (Qbiogene, http://www.qbiogene.com). The EGY48 transformants expressing pJG4–5 containing *RIN4* were selected on minimal SD glucose agar base supplemented with –Trp DO (Qbiogene). After plating, the plates were incubated for 3 to 4 d at 30 °C until colonies appeared.

Pairwise matings were set up between RFY206 (+pSH18–34):LexABD-avrB strains and EGY48:B42AD-RIN4 or EGY48:B42AD. The standard yeast mating procedure was followed according to the manufacturer's protocols (Clontech Yeast Protocols Handbook). A 100-μl aliquot of the mating culture was spread on SD Glucose Agar base supplemented with –Ura/–His/–Trp DO to select for diploids. Plates were incubated at 30 °C for 3 to 4 d to allow yeast cotransformants to form visible colonies. In the diploid strain, the two reporters are *LEU2* and *lacZ*. To assay for protein–protein interactions, yeast cotransformants were replica plated onto two different selective media containing galactose (Gal) to induce the expression of B42AD-RIN4 protein. Plates were incubated at 30 °C for 3 to 4 d until growth was detected.

1. SD Gal agar base supplemented with –Ura/–His/–Trp DO to confirm the nutritional phenotypes of the diploid by selecting for the LexABD, B42AD, and pSH18–34 vectors.

2. SD Gal agar base supplemented with –Ura/–His/–Trp/–Leu/X-Gal DO to screen for *Leu2* and *lacZ* reporter gene expressions. Growth and blue color were monitored based on activation of the reporter genes and were scored as a positive interaction between the fusion proteins. No interaction was scored if the replicates grew only on Gal/SD/–Ura/–His/–Trp DO plate and not on agar base supplemented with Gal/SD/–Ura/–His/–Trp/–Leu/X-Gal.

To determine LexABD-AvrB accumulation in yeast, RFY206 yeast cultures were grown in selective medium overnight. The cultures were diluted to an OD_600_ = 0.15 – 0.2. The cultures were continuously monitored until an OD_600_ of 0.4 to 0.6 was reached. The cells were pelleted and proteins were extracted and boiled in 50 mM sodium phosphate (pH 7.0), 25 mM 2-morpholinoethanesulfonic acid (MES) (pH 7.0), 3 M urea, 1% SDS, 10% β-mercaptoethanol (BME), and 0.1% Bromophenol Blue supplemented with Complete Protease Inhibitor Pellets (Roche). The boiled samples were spun briefly in the tabletop centrifuge to pellet cell debris. A volume equivalent to 0.5 total OD_600_ was loaded into each well on a 10% SDS-PAGE gel. After electrophoresis, the proteins were transferred from the SDS-PAGE gel to nitrocellulose membrane support. Western blots were done by standard methods. Anti-LexBD antibody was used at 1:100 (Santa Cruz Biotechnology, http://www.scbt.com). Detection of LexA-AvrB was with the goat monoclonal antibody. Detection of the peroxidase signal of the secondary antibody-HRP conjugate was performed with ECL (Amersham Biosciences).

### AvrRpt2 translocation assays.

Selected AvrB mutants were fused to Δ79AvrRpt2 by cloning into Gateway-compatible pBBR1-MCS2 [32,33] using LR clonase and transformed into Escherichia coli DH5α. Each construct was introduced in *Pto* strain DC3000 by triparental matings. Infiltrations of *Arabidopsis rpm1–3* were done as described [9]. The HR was scored 24 h after *Pto* DC3000 inoculations. Results were compared with leaves infiltrated with *Pto* carrying full-length *avrRpt2* or an empty vector.

## Supporting Information

Figure S1 AvrB Contacts with Main-Chain Residues of RIN4_142–176_
Antiparallel beta-strand hydrogen-bonding interactions between main-chain residues of RIN4_142–176_ with AvrB, as well as a hydrogen bond between the main-chain of the RIN4_142–176_ and the guanidinium group of R209 in AvrB. Stick representations of main-chain residues from RIN4_142–176_ are labeled green. Main-chain residues of AvrB contacting RIN4_142–176_ and the side-chain of R209 are labeled blue. AvrB helices and strands are red and blue, respectively.
(48 KB PDF)

Figure S2 Functional Consequence of Additional AvrB Mutations of RIN4_142–176_ Contact Residues and Residues in the ADP Binding Pocket (A) Quantification of RPM1-dependent mediated cell death by electrolyte leakage (mean ± 2 SE) of leaves infected with *Pto* DC3000 expressing wild-type AvrB-HA or AvrB-HA mutants at amino acids in the RIN4 binding groove. Positive and negative control infections with *Pto* DC3000(*avrB*-*HA*) or the *Pto* DC3000(EV) are represented by solid black lines and symbols as given, and mutants with dashed lines and symbols as given.
(B) Quantification of RPM1-dependent mediated cell death by electrolyte leakage (mean ± 2 SE) of leaves infected with *Pto* DC3000 expressing wild-type AvrB-HA or AvrB-HA mutants at amino acids lining the ADP binding domain, done as in (A). Note that N62A and F113A give intermediate levels of RPM1-mediated ion leakage.
(C) Trypan blue staining of *Arabidopsis* Col-0 leaves 5 h after infection with *Pto* DC3000 expressing wild-type or mutant versions of AvrB-HA. Experiments presented in (A through C) were repeated twice using ten leaves per AvrB allele for trypan blue staining and 12 leaves per AvrB allele for ion leakage experiments. Note that N62A and F113A give intermediate levels of RPM1-mediated trypan blue staining.
(D) Western blot analysis of soluble protein from *Pto* DC3000 expressing wild-type or various mutant versions of AvrB-HA, detected using anti-HA monoclonal antibody. Twenty micrograms of soluble protein from *Pto* DC3000 expressing each construct and grown in minimal media was loaded in each lane.
(191 KB PDF)

Figure S3 All Loss-of-Function AvrB Mutant Alleles Are Translocated into Plant Cells Type III–dependent secretion was confirmed for all loss-of-function AvrB mutant alleles. Wild-type AvrB or AvrB mutants as listed were independently expressed as N-terminal fusions to the C-terminal, HR-inducing domain of AvrRpt2 (AvrRpt2:80–255) in *Pto* DC3000, and inoculated onto Col-0 (*rpm1*) mutant plants. All *avrB::avrRpt2:80–255* strains triggered HR 20 h after infiltration. By contrast, expression of AvrRpt2:81–255 in the absence of AvrB (EV, empty vector control) did not induce RPS2-mediated HR (not shown).
(25 KB PDF)

### Accession Numbers

Crystallographic coordinates are deposited at the RCSB Protein Data Bank (http://www.rcsb.org/pdb) with the codes 2NUD and 2NUN for the AvrB/RIN4 and AvrB/ADP complexes, respectively; and 1CDK for inhibitor peptide in which the acceptor serine 17 is replaced by an alanine. The GenBank (http://www.ncbi.nlm.nih.gov/Genbank) accession numbers for the paralogs used in Figure 5 are AvrB (P13835), AvrC (P13836), AvrPphC (AAV68743), AvrB2 (YP_272229), AvrB4–1 (YP_275207), AvrB4–2 (YP_273068), AvrXccC (XCC2109), AvrB *Psy*1 (AAN85189), AvrB *Psy*2 (AAF71496), and AvrB3 (YP_275207).

## Abbreviations

ADP: adenosine diphosphate
ATP: adenosine triphosphate
AvrB: avirulence protein B
AvrC: avirulence protein C
AvrPto: avirulence protein *Pseudomonas* tomato
AvrRpm1: avirulence protein Rpm1
AvrRpt2: avirulence protein Rpt 2
AvrXccC: Xanthomonas campestris campestris avirulence protein C
BBS: AvrB binding site
EDTA: ethylenediaminetetraacetic acid
HR: hypersensitive cell death
ITC: isothermal titration calorimetry
NB-LRR: nucleotide-binding leucine-rich repeat
RCS: AvrRpt2 restrictive cleavage site
RIN4: RPM1-interacting protein
RPM1: resistance to Pseudomonas maculicula protein 1
RPS2: resistance to Pseudomonas syringae protein 2
TTSS: type III secretion system
