# Correction: Type III Effector Activation via Nucleotide Binding, Phosphorylation, and Host Target Interaction

**DOI:** 10.1371/journal.ppat.0030090

**Published:** 2007-06-29

**Authors:** Darrell Desveaux, Alex U Singer, Ai-Jiuan Wu, Brian C McNulty, Laura Musslewhite, Zachary Nimchuk, John Sondek, Jeffrey L Dangl

In *PLoS Pathogens,* volume 3, issue 3: doi:10.1371/journal.ppat.0030048

On page 0457, in the first paragraph of the Results section, the above paper states:

“Additionally, AvrB T182 contacts RIN4 Y151, and AvrB V128 contacts RIN4 D155, S161, and G162”.

This should read:

“Additionally, AvrB T182 contacts RIN4 F151, and AvrB V128 contacts RIN4 D155, S161, and A162”.

Further, in Table 2, line 2, G163 should be A162; and in Table 2, line 9, Y151 should be F151. The corrected table is as follows:

**Table 2:**
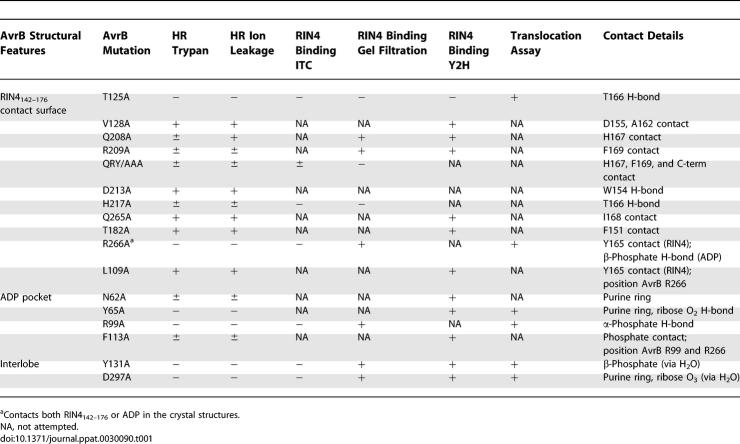
List of AvrB Mutations

The authors regret these errors and thank K. Caldwell and R. Michelmore for alerting us to them.

