# Supplementary figures and images for: Type III Effector Activation via Nucleotide Binding, Phosphorylation, and Host Target Interaction
Source: PLoS Pathog. 2007 Mar 30;3(3):e48. doi: 10.1371/journal.ppat.0030048 (PMC1839166; doi:10.1371/journal.ppat.0030048)

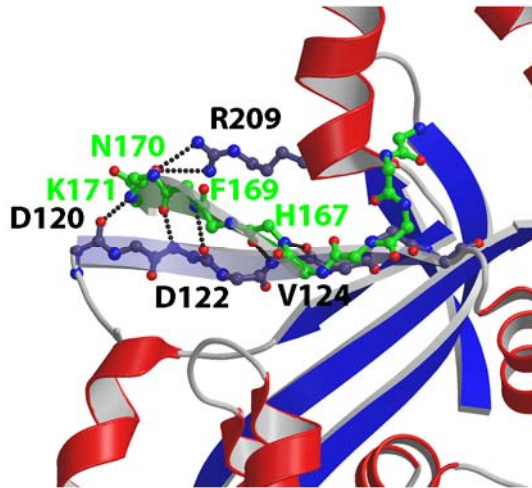

Supplement: Figure S1 — Antiparallel beta-strand hydrogen-bonding interactions between main-chain residues of RIN4142–176 with AvrB, as well as a hydrogen bond between the main-chain of the RIN4142–176 and the guanidinium group of R209 in AvrB. Stick representations of main-chain residues from RIN4142–176 are labeled green. Main-chain residues of AvrB contacting RIN4142–176 and the side-chain of R209 are labeled blue. AvrB helices and strands are red and blue, respectively. (48 KB PDF) [file ppat.0030048.sg001.pdf]

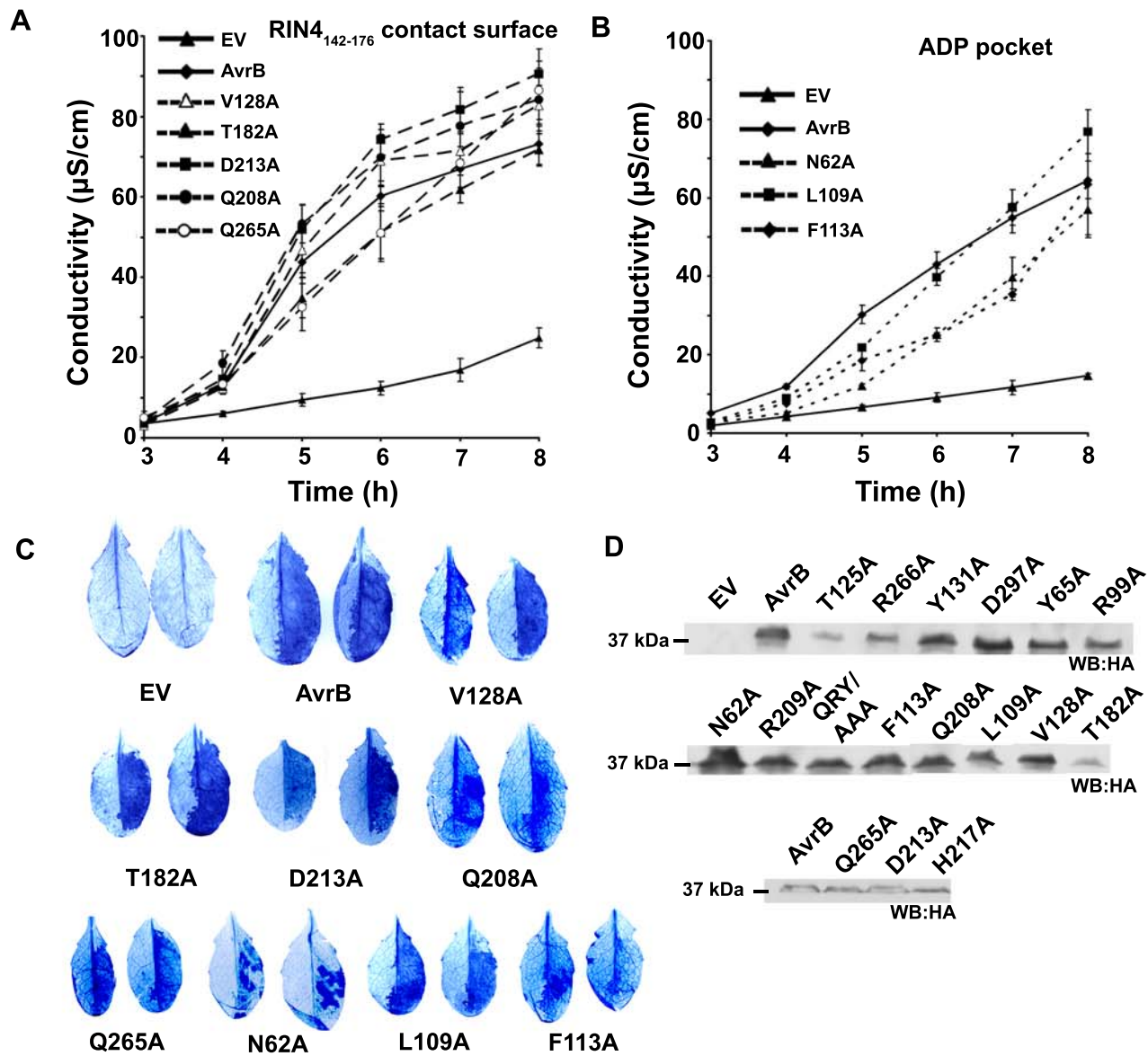

Supplement: Figure S2 — (A) Quantification of RPM1-dependent mediated cell death by electrolyte leakage (mean ± 2 SE) of leaves infected with Pto DC3000 expressing wild-type AvrB-HA or AvrB-HA mutants at amino acids in the RIN4 binding groove. Positive and negative control infections with Pto DC3000(avrB-HA) or the Pto DC3000(EV) are represented by solid black lines and symbols as given, and mutants with dashed lines and symbols as given. (B) Quantification of RPM1-dependent mediated cell death by electrolyte leakage (mean ± 2 SE) of leaves infected with Pto DC3000 expressing wild-type AvrB-HA or AvrB-HA mutants at amino acids lining the ADP binding domain, done as in (A). Note that N62A and F113A give intermediate levels of RPM1-mediated ion leakage. (C) Trypan blue staining of Arabidopsis Col-0 leaves 5 h after infection with Pto DC3000 expressing wild-type or mutant versions of AvrB-HA. Experiments presented in (A through C) were repeated twice using ten leaves per AvrB allele for trypan blue staining and 12 leaves per AvrB allele for ion leakage experiments. Note that N62A and F113A give intermediate levels of RPM1-mediated trypan blue staining. (D) Western blot analysis of soluble protein from Pto DC3000 expressing wild-type or various mutant versions of AvrB-HA, detected using anti-HA monoclonal antibody. Twenty micrograms of soluble protein from Pto DC3000 expressing each construct and grown in minimal media was loaded in each lane. (191 KB PDF) [file ppat.0030048.sg002.pdf]

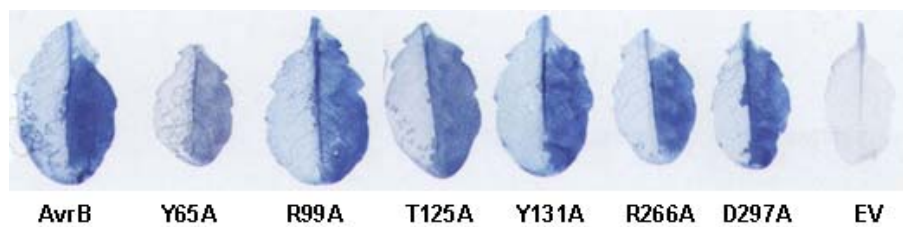

Supplement: Figure S3 — Type III–dependent secretion was confirmed for all loss-of-function AvrB mutant alleles. Wild-type AvrB or AvrB mutants as listed were independently expressed as N-terminal fusions to the C-terminal, HR-inducing domain of AvrRpt2 (AvrRpt2:80–255) in Pto DC3000, and inoculated onto Col-0 (rpm1) mutant plants. All avrB::avrRpt2:80–255 strains triggered HR 20 h after infiltration. By contrast, expression of AvrRpt2:81–255 in the absence of AvrB (EV, empty vector control) did not induce RPS2-mediated HR (not shown). (25 KB PDF) [file ppat.0030048.sg003.pdf]
